# Peptide-Mediated Liposomal Drug Delivery System Targeting Tumor Blood Vessels in Anticancer Therapy

**DOI:** 10.1155/2010/723798

**Published:** 2010-05-05

**Authors:** Han-Chung Wu, De-Kuan Chang

**Affiliations:** Institute of Cellular and Organismic Biology, Academia Sinica, Taipei 11529, Taiwan

## Abstract

Solid tumors are known to recruit new blood vessels to support their growth. Therefore, unique molecules expressed on tumor endothelial cells can function as targets for the antiangiogenic therapy of cancer. Current efforts are focusing on developing therapeutic agents capable of specifically targeting cancer cells and tumor-associated microenvironments including tumor blood vessels. These therapies hold the promise of high efficacy and low toxicity. One recognized strategy for improving the therapeutic effectiveness of conventional chemotherapeutics is to encapsulate anticancer drugs into targeting liposomes that bind to the cell surface receptors expressed on tumor-associated endothelial cells. These anti-angiogenic drug delivery systems could be used to target both tumor blood vessels as well as the tumor cells, themselves. This article reviews the mechanisms and advantages of various present and potential methods using peptide-conjugated liposomes to specifically destroy tumor blood vessels in anticancer therapy.

## 1. Introduction

One of the primary goals of a successful cancer treatment regimen is to deliver sufficient amounts of drug to tumors while minimizing damage to normal tissues. Most chemotherapeutic agents enter normal tissues in the body with indiscriminate cytotoxicity and do not preferentially accumulate at tumor sites. At times the dose reaching the tumor may be as little as 5% to 10% of the doses accumulating in normal organs [1, 2]. One reason for the inability for drugs to accumulate at target sites is that the interstitial fluid pressure (IFP) in solid tumors is higher than in normal tissues, that blocking transcapillary transport of chemotherapeutic drugs or antibodies [3–5]. In this way, the anticancer effect is decreased and toxic effect to normal cells is increased. Fear of severely harming the patients often limits the dose of anticancer drugs that can be given to a patient. These lower than optimal doses elicit incomplete tumor responses which leads to disease relapse and drug resistance. Therefore, most cancer drugs fail in clinical studies not because they are ineffective in killing cancer cells but because they cannot be administered in doses high enough to eradicate the tumor without severely harming the patient. 

Several approaches have been developed to improve the ability of anticancer drug to more specifically target tumors and avoid normal organs. One of the most effective strategies is to encapsulate drugs in particles that deliver them preferentially to tumor sites. For example, liposome particles have been found able to deliver radionuclides, genes, and chemotherapeutic agents to tumor sites. [6–10]. Another promising strategy is to encapsulate anticancer drugs in liposomes conjugated with moieties, such as antibodies and peptides, that target particular types of target tumor cells or tumor vasculatures [11–13]. Use of internalizing ligands for targeting liposomes conjugated with such moieties makes it possible to deliver the chemotherapeutic drugs encapsulated within them to the cytosol through the receptor-mediated endocytosis [14–17]. This article reviews the current research in developing liposomal drug delivery systems that use peptide ligands to target blood vessels in solid tumors. We discuss the identification of peptides that can target tumor blood vessels and the use of targeting and nontargeting liposomes to encapsulate and deliver chemotherapeutic drugs to tumor sites.

## 2. Inhibiting Angiogenesis

Virtually every conventional cytotoxic drug has been found to be antiangiogenic in in vitro and in vivo models [18]. One treatment approach known as metronomic therapy uses frequent administrations of low-dose antiangiogenic agents to destroy vessels in tumors while decreasing the toxicity to normal tissues [19–21]. For example, it has been found in mice that frequent administration of relatively low, noncytotoxic doses of liposome-encapsulated doxorubicin can shrink various solid tumor xenografts [13, 16]. The antiangiogenic agent bevacizumab (Avastin), a humanized monoclonal antibody against vascular endothelial growth factor (VEGF), has been used with some success to treat advanced colon cancer. One study compared the effect using three chemotherapeutic agents alone to treat advanced colon cancer with using the three agents combined with bevacizumab [22]. They found that the combined use of chemotherapeutic agents and bevacizumab extended overall survival by approximately 4.7 months compared to the use of chemotherapeutic agents alone [22]. Other angiogenesis inhibitors, including sunitinib and sorafenib, have also been found to improve clinical outcomes when used to treat various cancer types [23, 24].

The targeting of proliferating endothelial cells in the blood vessels of tumors has several advantages. First, endothelial cells in malignant tumors are genetically stable, nonmalignant, and rarely drug resistant, compared to the cancer cells [19, 21]. However, some recent studies show that tumor-associated endothelial cells can acquire cytogenetic abnormalities while they are in the tumor microenvironment [25, 26]. Second, the destruction of endothelial cells using this method amplifies the drugs antitumor effect. It has been reported that the elimination of one endothelial cell can inhibit the growth of as many as a hundred tumor cells [27, 28]. Third, antiangiogenic therapy decreases IFP within the tumor allowing better penetration by chemotherapeutic agents [29–32]. For example, Jain found that bevacizumab could decrease IFP by normalizing tumor vasculature and decreasing vascular leakage [29, 33]. Fourth, antiangiogenic therapy is known to inhibit the growth of both primary and metastatic solid tumors. Finally, intravenously injected angiogenesis inhibitors can directly reach endothelial cells.

In addition, we can take advantage of the differences between endothelial cell plasma membrane proteins (i.e., vascular zip codes) to develop drug delivery systems capable of guiding therapeutic or imaging agents to a particular organ or tumor [34, 35]. Endothelial cells of blood vessels within solid tumors express certain molecular structures that are absent or minimally detectable in normal blood vessels [13, 36, 37]. These structures can be used as molecular targets for antitumor treatment. 

## 3. Identifying Peptides That Target Tumor Blood Vessels

The key to delivering drugs specifically to these targets is to identify and use ligands that specifically bind to and that can be internalized by endothelial cells in tumors. Combinatorial peptide libraries displayed on microorganisms have become a research tool for identifying cell surface-binding peptides that can become targets for antitumor treatment. Of the many molecular display techniques, phage display has been the most popular approach. Phage display is a selection technique in which a peptide or protein is fused with a coat protein of bacteriophage and displayed on the surface of the virion. Phage-displayed random peptide libraries have helped researchers map B-cell epitopes [38–40], discover protein-protein contacts [41, 42], and identify bioactive peptides bound to receptors [43, 44] or proteins [45, 46]. Peptide libraries can be used to find disease-specific antigens [47, 48] and cell- [2, 49] and organ-specific peptides [16, 35, 36]. 

**Figure 1:**
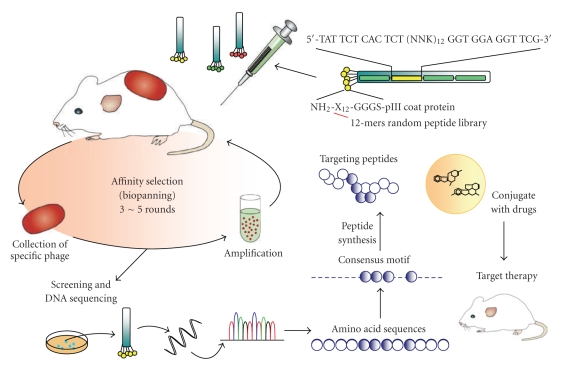
Selection of peptides that target tumor blood vessels using in vivo phage display. Peptide or antibody libraries are expressed as fusion proteins with a coat protein (pIII) of a bacteriophage, and the fused proteins are displayed on the surface of the virion. A phage-displayed peptide library was injected through the tail vein of tumor-bearing mice. Eight minutes after injection, the mice were perfused through the heart. Phage recovered from the tumor was amplified and reinjected in mice for another four rounds. Tumor-targeting phages were further identified by in vivo tumor-homing assay, synthetic peptide binding and competition assay, and immunohistochemical staining. The identified peptides can be used as ligands to recognize cell surface markers or tumor antigens to develop targeted therapy. SCID mice bearing human cancer xenografts were successfully treated with ligand-conjugated antiangiogenic targeting liposomes.

Recently, using affinity selection (biopanning) of phage-displayed peptide libraries, researchers have discovered molecules that are expressed on tumor blood vessels exclusively [16, 34–36]. The strategy for identifying tumor-targeting ligands and developing ligand-mediated targeted therapy is shown in Figure 1. Researchers have used in vivo affinity selection of phage libraries to identify peptides that interact with the molecules found on endothelia in tumors [34, 36]. The NGR peptide motif targets angiogenic blood vessels [36] and the tumor-homing property of NGR motif relies on recognition of a CD13 isoform selectively expressed within tumor blood vessels [54]. Compared with the nontargeting liposomal doxorubicin (Caelyx), NGR peptide-conjugated Caelyx significant improvements in survival was seen in clinically relevant animal models of neuroblastoma, ovarian, and lung cancers [17]. Another peptide, SP5-52, has been found to recognize blood vessels created in tumors but not normal blood vessels in severe combined immunodeficiency (SCID) mice bearing solid tumors. Several selected phage clones display Pro-Ser-Pro, a motif crucial to peptide binding to tumor neovasculature [13]. Several tumor homing peptides have been found to bind to blood vessels in surgical specimens of human cancer and they have also been found to home to tumor tissues of different human tumor xenografts as confirmed by in vivo homing assays [16]. These studies found a greater correlation between increased tumoral accumulation of the targeting liposomes and antitumor efficacy than the accumulation of free drugs or drugs formulated in the nontargeting liposomes [2, 13, 16].

## 4. Drug-Encapsulated Liposomes

Most of the drug delivery systems approved for marketing are liposomal- or lipid-based formulations or therapeutic molecules linked to polyethylene glycol (PEG) [6, 10, 55, 56]. One such product is PEGylated liposomal doxorubicin, which is known as Doxil in the US and Caelyx in Europe [57]. It is currently approved for the treatment of AIDS-related Kaposi's sarcoma and recurrent ovarian cancer in North America, Europe, and other countries, and for metastatic breast cancer in Europe. Liposome-encapsulated doxorubicin has been found to significantly improve the therapeutic index of doxorubicin both in preclinical [58–60] and in clinical studies [61–64]. An important advantage of PEGylated liposomal doxorubicin is that the heart muscle uptakes much less of it than free doxorubicin [58, 65]. One study found no cardiotoxicity in 40 patients receiving cumulative doses of 500–1500 mg/m^2^ of doxorubicin [62]. Free doxorubicin, on the other hand, is limited to a maximum recommended cumulative dose of 450–550 mg/m^2^. Colbern et al. found that the activity of PEGylated liposomal doxorubicin 1-2 mg/kg was almost equivalent to that of free doxorubicin 9 mg/kg in mouse Lewis lung carcinoma [59]. One clinical study reported that most (>98%) of the drug circulating in the blood stream remains in encapsulated in liposomes [61], suggesting that little of the liposomal drugs will be leaked to the circulation system during its journey to the tumor tissues.

The hyperpermeability of tumor vasculature is a key factor for the success of liposome-delivered chemotherapy agents. The “leakiness” of the angiogenic tumor vasculature is estimated to have an average pore size of 100–600 nm [66]. These pores are significantly larger than the gap junction found in normal endothelium, which are typically <6 nm wide [67]. Liposomes with diameters of approximately 65–75 nm [13, 14, 50] are small enough to passively infiltrate tumor endothelium but large enough to be excluded from normal endothelium. Hence, they selectively extravasate into the tumor interstitial space. In the tissue of solid tumors, vasculature becomes so permeable that particulate liposomes can extravasate and localize in the tissue interstitial space [6, 10]. In addition, tumor tissues frequently lack effective lymphatic drainage [3], which means that the liposomes can be retained longer. Together, these factors increase the accumulation of the drug within the tumor, which has been referred to as the “enhanced permeability and retention (EPR) effect” by Maeda et al. [68, 69]. EPR-mediated passive tumor targeting by liposomes can increase the concentration of drugs in solid tumors by as much as ten times, compared to free drugs [70]. 

Passively targeted liposomal drug delivery systems have some disadvantages. Normal organ uptake of liposomes leads to accumulation of the encapsulated drug in mononuclear phagocytic system cells in the liver, spleen, and bone marrow [63], which may present hazards to these tissues. For example, with increased circulation time of these drugs may come increased toxicity inducing such problems as hand-foot syndrome, mucositis, and hematological toxicities such as neutropenia, thrombocytopenia, and leucopenia [71–74]. Therefore, ongoing research aims at enhancing the tumor site-specific action of the liposomes by attaching ligands to surface molecules of tumor cells and tumor vasculature, a process called active or ligand-mediated targeting liposomes [5, 6, 13, 75].

## 5. Peptide-Mediated Targeting Liposomes

The disadvantage of the passive PEGylated liposomes can be overcome by creating ligand-mediated targeting liposomes with more selective anticancer activity. The activity of anticancer drugs can be enhanced by coupling targeting moieties to the surface of liposomes to promote selective binding to tumor-associated antigens and facilitate the delivery of drug-containing liposomes to the intended cellular sites. This drug delivery system has a higher drug-to-carrier ratio than immunoconjugates and multivalent presentation of ligands, which increases their binding avidity [11]. 

Antibodies that bind to tumor-specific antigens have so far yielded little success as a drug delivery system for solid tumors, which make up more than 90% of all cancers in humans. Although monoclonal antibodies have shown clinical potential as tumor targeting agents, they are limited by their large molecular size and poor tumor penetration [76], by the immunogenicity associated with immunoliposomes, and by their toxicity to liver and bone marrow from nonspecific antibody uptake. These limitations can be overcome by using peptide ligands, which are smaller, less immunogenic molecules, and easier to produce and manipulate. Furthermore, peptide ligands have moderate affinity to antigens, which is beneficial because extremely high affinity of antibody-binding can impair tumor penetration [77]. Compared with antibody ligands, peptide ligands can improve tumor penetration and decrease MPS clearance of conjugated liposomes [50, 78]. The increasing use of peptides as targeting ligands has been aided by the use of phage display to identify novel ligands (Figure 1). Researchers have already produced liposomes conjugated with ligands that specifically target tumor cells or tumor vasculature [5, 16, 17].

**Figure 2:**
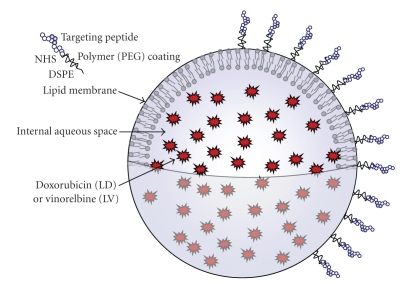
Generation of peptide-conjugated liposomes targeting tumor blood vessels. A single lipid bilayer membrane separates an internal aqueous compartment from the external medium. Doxorubicin was encapsulated in the internal compartment. Drug molecules are tightly packed (10,000 to 15,000 molecules per liposome) in a gel phase. Tumor-homing peptide ligands were coupled to NHS-PEG-DSPE [N-hydroxysuccinimido-carboxyl-polyethylene glycol-derived distearoylphosphatidylethanolamine] in a 1 : 1.5 molar ratio [13, 14, 50]. The reaction was completed and confirmed by quantifying the remaining amino groups using TNBS (Trinitrobenzenesulfonate) reagent [51]. Peptidyl-PEG-DSPE was transferred to preformed liposomes after coincubation at a temperature above the transition temperature of the lipid bilayer [52]. There were 500 peptide molecules per liposome [53]. The mean diameter of the targeting liposome is approximately 75 nm [2, 13].

Peptide-conjugated liposomes have three main components: anticancer drug, a liposome carrier, and targeting peptide (Figure 2). Remote loading methods such as the ammonium sulfate method [13, 79] and the pH gradient method [80] can encapsulate weak bases such as doxorubicin or vinorelbine into the liposomes with more than 95% efficiency. Schedule-dependent drugs such as vinca alkaloids, topotecan, and 5-fluorouracil are also potential candidates for liposomal delivery because they can extend the time when cancer cells are exposed to therapeutic levels of the drug. 

**Figure 3:**
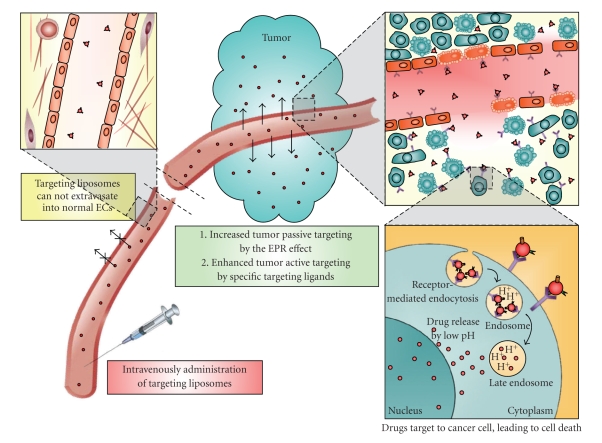
Diagram of the molecular mechanism of peptide-conjugated liposomes on cancer therapy. These liposomes prolong circulation time in blood and improve pharmacokinetic and biodistribution of their encapsulated drugs. After intravenous administration, liposomes are large enough to be excluded from normal endothelium. In solid tumors, the angiogenic tumor vasculature becomes leakiness that particulate liposomes can extravasate and localize in the tissue interstitial space making it possible for more drug delivering liposomes to accumulate within the tumor by EPR effect. Coupling liposomes with peptides targeted to tumor cells or tumor vasculature further enhances the specificity and accumulation of liposomes in the tumor. On arrival in the tumor tissues, the liposomes are bound and internalized by tumor cells or tumor-associated endothelial cells through receptor-mediated endocytosis, fused with the low pH compartments of the endosomes, and subsequently broken down the liposomes and to release encapsulated drugs into the intracellular space of the cells.

The bioavailability and pharmacodynamics of liposome-encapsulated chemotherapeutic drugs must be considered in developing these delivery systems. To take advantage of the EPR effect, liposomes need to have long half-lives so that the drug stays within the carrier as long as possible in blood circulation until it accumulates in diseased tissues [81]. Once liposomes are localized to a solid tumor, the drug they contain must be released and become bioavailable at a rate remains therapeutically effective for a period of time. The rate of active drug's release into tumor cells, not the total drug concentration in the tumor tissues, is critical for measuring the actual bioavailability of the liposomal drug [16]. Some targeting liposomes have not been found to have greater therapeutic efficacy than passive liposomal drugs, possibly because the lack of internalizing ligands does not give the drug greater access inside tumor cells [82, 83]. Drug delivery can be further enhanced if the liposome-attached ligands bind selectively to internalizing antigens which would help increase the concentration of drugs inside tumor or tumor-associated endothelial cells resulting in higher drug concentration inside the cells [13, 15, 84, 85]. This binding to internalizing antigens by ligands can induce receptor-mediated endocytosis of liposomes into endosome compartments with low pH, where the liposomes break down and release the encapsulated drug into the intracellular space (Figure 3). These steps lead to higher intracellular drug concentration and greater destruction or inhibition of tumor cells. Studies have confirmed greater cytotoxic effects produced by liposomes with peptides that target internalizing antigens through enhanced specificity and improved drug bioavailability [2, 16]. 

The use of drug-encapsulated liposomes with ligands to target tumor blood vessels allows us to destroy both tumor blood vessels and tumor cells. In mice bearing human cancer xenograft, low dose of peptide-conjugated liposomal doxorubicin has been found to markedly inhibit vascularization and reduce total volume and weight of tumors [13, 16, 17]. The immunofluorescent analysis of the tumors in several studies has revealed an association between significant decreases in microvessel density and increases in the apoptosis of tumor cells and tumor-associated endothelial cells. The severe damage to tumor vasculature caused by peptide-conjugated liposomal doxorubicin throughout the tumors suggested an improvement in chemotherapeutic efficacy over nontargeting liposomes and conventional drugs [13, 16, 17]. This dual action may produce a greater, more durable anticancer effect than is found with the use of simple antiangiogenic therapy. 

One peptide-conjugated liposome can deliver over ten thousand anticancer drug molecules directly into target tumor cells efficiently and effectively. The targeted and sustained release of the drug molecules can increase the maximum tolerated dose (MTD) of the cytotoxic drugs and dramatically lower dose-limiting toxicities, and in turn prevent treatment delay or discontinuation. The affinity of targeting ligands may allow the liposomes to move past the high IFP barrier within tumors [4, 5, 13, 16]. 

Advances in nanotechnology and molecular biology are moving us closer to developing an ideal “multifunctional smart nanodrug delivery system” using various types of ligands and drugs based on the kinds of diagnosis, imaging, or therapy needed. Such smart nanodrug delivery systems will allow accurate, specific, and noninvasive disease treatment, early diagnosis, and monitoring. In the future, combining ligands that specifically bind to cancer cells (including cancer stem cells) and tumor blood vessels with multifunctional liposomal drug delivery systems may help improve the effectiveness of cancer treatment and minimize the side effects traditionally associated with chemotherapy.

## 6. Conclusions

The development of highly selective anticancer drugs that can discriminate between tumor cells and normal cells is the most important goal of current oncology research. The potential use of ligand-conjugated liposome-encapsulated drugs to target tumor cells and vasculature is very promising. Peptides that specifically bind to tumor targets can be coupled to the PEG terminus of sterically stabilized liposomes and subsequently precisely deliver chemotherapeutic agents to tumor cells or blood vessels. Peptide-mediated liposomes that target vasculature are a new generation of chemotherapy delivery systems with superior pharmacokinetics, controlled biodistribution, efficacy, and safety profiles.
